# Supplementary material for: Skeletal muscle loss and body composition in progressive supranuclear palsy: A retrospective cross-sectional study
Source: PLoS One. 2021 Jun 10;16(6):e0253079. doi: 10.1371/journal.pone.0253079 (PMC8192011; doi:10.1371/journal.pone.0253079)
Supplement: Attachment — Submitted filename: Responce_to_Reviews_Takamatsu and Aiba_Plos One_21Apr2021.docx [file pone.0253079.s001.docx]

To editor and reviewers

We thank the editor and reviewers for taking the time to review our manuscript. We have revised the manuscript according to the editor’s and reviewers’ comments. The revised portions of the manuscript are highlighted in red.

**Additional Editor Comments:**

***Please consider citing in the introduction the following article, published by PLOS ONE, regarding the use of sarcopenia as predictor of survival also in the setting of cancer***

***https://journals.plos.org/plosone/article/comments?id=10.1371/journal.pone.0232371***

Following the editor’s suggestion, we cited the following article in the introduction: Lanza E et al. Sarcopenia as a predictor of survival in patients undergoing bland transarterial embolization for unresectable hepatocellular carcinoma. PLoS One. 2020.

Introduction: page 3, lines 44-45.

Sarcopenia has been associated with motor functional disability, lower quality of life (QOL), and mortality [2–4].

References: page 11, lines 233-236.

4. Lanza E, Masetti C, Messana G, Muglia R, Pugliese N, Ceriani R, et al. Sarcopenia as a predictor of survival in patients undergoing bland transarterial embolization for unresectable hepatocellular carcinoma. PLoS One. 2020;15: 1–12. doi:10.1371/journal.pone.0232371

**Journal Requirements:**

***Please review your reference list to ensure that it is complete and correct. If you have cited papers that have been retracted, please include the rationale for doing so in the manuscript text, or remove these references and replace them with relevant current references. Any changes to the reference list should be mentioned in the rebuttal letter that accompanies your revised manuscript. If you need to cite a retracted article, indicate the article’s retracted status in the References list and also include a citation and full reference for the retraction notice.***

We confirmed our reference list to be complete and correct.

***When submitting your revision, we need you to address these additional requirements.***

***1. Please ensure that your manuscript meets PLOS ONE's style requirements, including those for file naming. The PLOS ONE style templates can be found at***

***https://journals.plos.org/plosone/s/file?id=wjVg/PLOSOne_formatting_sample_main_body.pdf and***

***https://journals.plos.org/plosone/s/file?id=ba62/PLOSOne_formatting_sample_title_authors_affiliations.pdf***

Following the journal requirements, we revised the formatting of our manuscript based on these sample PDFs.

***2. Thank you for stating the following in the Competing Interests section:***

***"I.A. serves as a consultant for Biogen MA Inc. and AbbVie GK."***

***Please confirm that this does not alter your adherence to all PLOS ONE policies on sharing data and materials, by including the following statement: "This does not alter our adherence to PLOS ONE policies on sharing data and materials.” (as detailed online in our guide for authors http://journals.plos.org/plosone/s/competing-interests). If there are restrictions on sharing of data and/or materials, please state these. Please note that we cannot proceed with consideration of your article until this information has been declared.***

***Please include your updated Competing Interests statement in your cover letter; we will change the online submission form on your behalf.***

***Please know it is PLOS ONE policy for corresponding authors to declare, on behalf of all authors, all potential competing interests for the purposes of transparency. PLOS defines a competing interest as anything that interferes with, or could reasonably be perceived as interfering with, the full and objective presentation, peer review, editorial decision-making, or publication of research or non-research articles submitted to one of the journals. Competing interests can be financial or non-financial, professional, or personal. Competing interests can arise in relationship to an organization or another person. Please follow this link to our website for more details on competing interests: http://journals.plos.org/plosone/s/competing-interests***

Yes, we agree with the PLOS ONE policies on sharing data and materials. We mentioned about COI in cover letter.

***3. We note you have included a table to which you do not refer in the text of your manuscript. Please ensure that you refer to Table 2 in your text; if accepted, production will need this reference to link the reader to the Table.***

Following the journal requirements, we revised the manuscript.

Results: page 7, line 144.

In the entire PSP group, there was no significant correlation between SMI on one hand and disease duration, mRS and BI on the other (Table 2).

***Reviewers' comments:***

***Reviewer #1: I thank the authors for taking consideration of the relation between body composition parts and PSP patients. In order for the manuscript to be scientifically valid regarding the association suggested, I suggest that adjustment for other confounders causing wasting and sarcopenia should be mentioned in the manuscript [exercise state of the patient versus controls, the caloric pattern [moderate, mild or high according to daily activity and occupation], details of ergogenic or ergopenic medications and nutritional state including the presence or absence of bulbar dysfunction or anorexia]. These factors should be elucidated in my experience in order to make the publication worthy of targeting the association between PSP and sarcopenia.***

Thank you for raising very important points. The remark about confounders causing sarcopenia is critical and the lack of data was a significant limitation in this study. Therefore, following the reviewer’s comment, we revised the manuscript and added some references.

Discussion: page 9, lines 185-189.

The second limitation was that this study was lack of data about related factors and confounders causing skeletal muscle mass loss or sarcopenia, such as motor function (e.g., muscle strength, gait speed) [1,15], daily physical activity (e.g., exercise state) [20,21], nutrition sate (e.g., caloric pattern, intake of ergogenic or ergogenic drugs, bulbar dysfunction, anorexia) [22–25].

References: page 13, line 294 – page 14, line 312.

20.	Steffl M, Bohannon R w, Sontakova L, Tufano JJ, Shiells K, Holmerova I. Relationship between sarcopenia and physical activity in older people: a systematic review and meta-analysis. Clin Interv Aging. 2017;Volume 12: 835–845. doi:10.2147/CIA.S132940

21.	Lee SY, Tung HH, Liu CY, Chen LK. Physical Activity and Sarcopenia in the Geriatric Population: A Systematic Review. J Am Med Dir Assoc. 2018;19: 378–383. doi:10.1016/j.jamda.2018.02.003

22.	Naseeb MA, Volpe SL. Protein and exercise in the prevention of sarcopenia and aging. Nutr Res. 2017;40: 1–20. doi:10.1016/j.nutres.2017.01.001

23.	Cruz-Jentoft AJ, Kiesswetter E, Drey M, Sieber CC. Nutrition, frailty, and sarcopenia. Aging Clin Exp Res. 2017;29: 43–48. doi:10.1007/s40520-016-0709-0

24.	Azzolino D, Damanti S, Bertagnoli L, Lucchi T, Cesari M. Sarcopenia and swallowing disorders in older people. Aging Clin Exp Res. 2019;31: 799–805. doi:10.1007/s40520-019-01128-3

25.	Zhao WT, Yang M, Wu HM, Yang L, Zhang X mei, Huang Y. Systematic Review and Meta-Analysis of the Association Between Sarcopenia and Dysphagia. J Nutr Heal Aging. 2018;22: 1003–1009. doi:10.1007/s12603-018-1055-z

***Reviewer #2: Manuscript by Takamatsu and Aiba is the first to report skeletal muscle loss in progressive supra nuclear palsy (PSP). Small size of studied groups of patients and controls is a significant limitation. However comparative analyses performed by sex, identified significant decrease in SMI in PSP groups driven by leg SMI.***

***This paper should be of interest in the field of clinical PSP.***

Thank you for reviewing our manuscript and being interest. We would like to increase the sample size and proceed with further verification.
